# Rapid and sensitive detection of methicillin-resistant *Staphylococcus aureus* through the RPA-*Pf*Ago system

**DOI:** 10.3389/fmicb.2024.1422574

**Published:** 2024-08-21

**Authors:** Weizhong Chen, Jiexiu Zhang, Huagui Wei, Jie Su, Jie Lin, Xueyan Liang, Jiangtao Chen, Rong Zhou, Lin Li, Zefang Lu, Guangyu Sun

**Affiliations:** ^1^Chaozhou People’s Hospital, Shantou University Medical College, Chaozhou, China; ^2^Department of Histology and Embryology, Shantou University Medical College, Shantou, China; ^3^School of Laboratory Medicine, Youjiang Medical University for Nationalities, Baize, China; ^4^Department of Laboratory, Chaozhou Central Hospital, Chaozhou, China; ^5^Department of Laboratory, Huizhou Central Hospital, Huizhou, China

**Keywords:** *Pf*Ago, RPA, POCT, nucleic acid detection, *Staphylococcus aureus*, methicillin-resistant *Staphylococcus aureus*

## Abstract

**Introduction:**

Both the incidence and mortality rates associated with methicillin-resistant *Staphylococcus aureus* (MRSA) have progressively increased worldwide. A nucleic acid testing system was developed in response, enabling swift and precise detection of *Staphylococcus aureus* (*S. aureus*) and its MRSA infection status. This facilitates improved prevention and control of MRSA infections.

**Methods:**

In this work, we introduce a novel assay platform developed by integrating *Pyrococcus furiosus* Argonaute (*Pf*Ago) with recombinase polymerase amplification (RPA), which was designed for the simultaneous detection of the *nuc* and *mecA* genes in MRSA.

**Results:**

This innovative approach enables visual MRSA detection within 55 mins, boasting a detection limit of 10^2^ copies/μL. Characterized by its high specificity, the platform accurately identifies MRSA infections without cross-reactivity to other clinical pathogens, highlighting its unique capability for *S. aureus* infection diagnostics amidst bacterial diversity. Validation of this method was performed on 40 clinical isolates, demonstrating a 95.0% accuracy rate in comparison to the established Vitek2-COMPACT system.

**Discussion:**

The RPA-*Pf*Ago platform has emerged as a superior diagnostic tool, offering enhanced sensitivity, specificity, and identification efficacy for MRSA detection. Our findings underscore the potential of this platform to significantly improve the diagnosis and management of MRSA infection.

## Graphical abstract

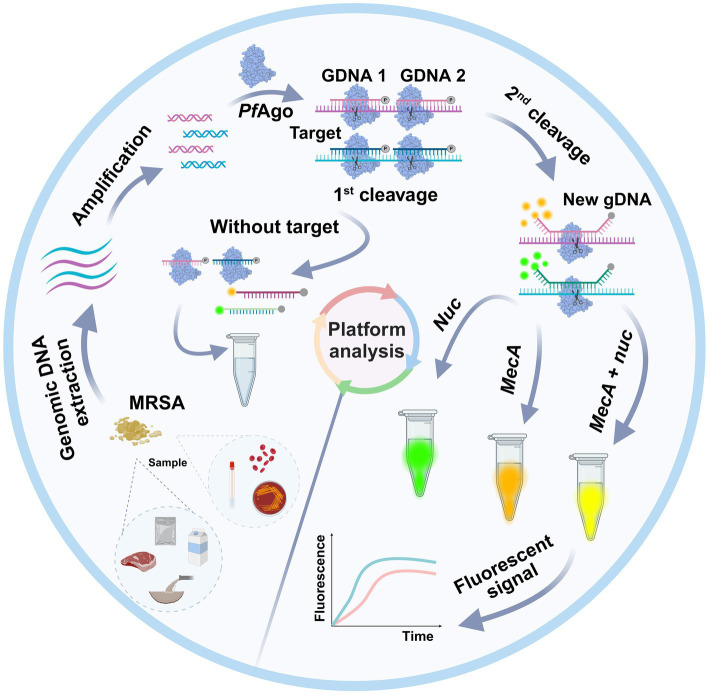

## 1 Introduction

*Staphylococcus aureus*, a ubiquitous gram-positive bacterium, colonizes human skin and mucous membranes and is emerging as a leading opportunistic pathogen (Gherardi, 2023; Linz et al., 2023). It is associated with a spectrum of diseases ranging from skin and soft tissue infections to severe respiratory tract infections and sepsis (GBD 2019 Antimicrobial Resistance Collaborators, 2022; Kouijzer et al., 2023; Li et al., 2023b). The revolutionary impact of penicillin and subsequent antimicrobial agents such as methicillin has transformed the treatment of bacterial infections. However, the rampant misuse and overuse of these drugs have led to the emergence of MRSA, which has posed a significant challenge to global health since its first identification in 1960 (Brakstad and Maeland, 1997; Harkins et al., 2017). MRSA resistance to β-lactam antibiotics has not only complicated the clinical management of *S. aureus* infections but also increased the risks involved (Arêde et al., 2012). The COVID-19 pandemic has significantly influenced the dynamics of antimicrobial resistance, particularly by increasing the incidence of hospital-acquired methicillin-resistant *Staphylococcus aureus* (HA-MRSA) infections. Research indicates that there was a 13% increase in the rate of HA-MRSA infections in 2020 compared with that in the previous year (Singulani et al., 2022; Lade and Kim, 2023). Additionally, MRSA poses a significant challenge to public health due to its rapid transmission and high risk of nosocomial cross-infection (Wang et al., 2023), leading to considerable strain on healthcare infrastructures (Tong et al., 2015). Consequently, there is a critical need for the development of an efficient and accurate pathogen detection system that can swiftly identify MRSA infections among patients. Implementing such a system is crucial for facilitating early diagnosis, enabling personalized treatment strategies, reducing patient suffering and death rates, and enhancing efforts to limit the spread of MRSA, ultimately reducing its impact on healthcare systems.

On the basis of antibiotic susceptibility, methicillin resistance in *S. aureus* is defined as an oxacillin minimum inhibitory concentration (MIC) greater than or equal to 4 micrograms/mL (Siddiqui and Koirala, 2024). Currently, MRSA detection mainly depends on traditional phenotypic and genotypic methods. The Clinical and Laboratory Standards Institute (CLSI M100-S24) recommends the use of conventional phenotypic assays such as paper diffusion, agar screening, and broth dilution for MRSA screening (Palavecino, 2020; Feng et al., 2023). These methods, however, require 2 to 3 days for incubation and involve complex procedures. To overcome these limitations, genotypic techniques such as polymerase chain reaction (PCR) have become the preferred standard for detecting MRSA by targeting specific genes, including *nuc* and *mecA* (Domann et al., 2000; Tenover and Tickler, 2022).

Additionally, other molecular detection strategies, such as real-time fluorescence quantitative PCR, nucleic acid molecular hybridization, and isothermal amplification, have been explored (Holfelder et al., 2006; Mutonga et al., 2019; Kim et al., 2022). Notably, recombinase polymerase amplification (RPA) is unique because of its simplicity, speed, and ability to operate at lower temperatures without complex thermal cycling, facilitating prompt detection (Kersting et al., 2014). Recent studies suggest that RPA-*Pf*Ago can achieve rapid, sensitive detection through various assays and genetic typing (Zhao et al., 2022; Liu et al., 2023b), indicating potential directions for future research to develop faster, more sensitive diagnostic tools for MRSA management.

CRISPR/Cas9 nucleic acid detection technology, known for its endonuclease activity, has gained significant attention as a powerful gene editing tool in recent years (Barazesh et al., 2021). Scientists have not only identified a variety of natural CRISPR–Cas9 systems (e.g., Cas9, Cas12a, and Cas12f) suitable for eukaryotic DNA editing but also developed novel CRISPR–Cas systems (e.g., Cas13a, Cas13b, and Cas13d) with improved efficiency and specificity for precise gene modification (Liu et al., 2022). These diverse CRISPR–Cas nucleases and their engineered variants have been widely used in biological research. Recent innovative assays, such as single-tube RPA-Cas12a/Cas13a for MRSA detection (Liu et al., 2023a) and LAMP-Cas12a technology, have demonstrated potential (Cao et al., 2023), as they can be implemented in portable devices and reduce the risk of aerosol contamination. Despite its high sensitivity and specificity, the CRISPR/Cas system still has certain limitations. In particular, unlike Ago-based methods, which offer greater versatility and accessibility by not depending on specific sequence motifs, CRISPR/Cas systems rely heavily on the PAM/PFS family (Ye et al., 2022). Moreover, the majority of guide RNAs needed for Cas effector proteins are either obtained through transcriptional purification or chemically synthesized *in vitro*, presenting challenges for multiplexed assays, increasing costs, and introducing instability, thereby restricting the diagnostic applications of CRISPR methods (Li et al., 2023a). In contrast, *Pf*Ago proteins have emerged as promising next-generation nucleic acid detection platforms due to their ability to precisely identify and cleave target sequences (Wang et al., 2021; Ye et al., 2022), offering an appealing alternative to address the challenges associated with the CRISPR–Cas system.

A highly efficient MRSA detection system was developed through the integration of RPA and *Pf*Ago technologies. The primary aim of this system is to achieve precise detection of both *S. aureus* and MRSA strains. To achieve this goal, specific markers for MRSA, namely, the *mecA* gene and *nuc* gene, were deliberately selected. The *mecA* gene, which is integral to methicillin resistance in *S. aureus*, encodes penicillin-binding protein 2a (PBP2a). This enzyme has a reduced affinity for β-lactam antibiotics, conferring resistance to these drugs (Hartman and Tomasz, 1984). On the other hand, the *nuc* gene, which is unrelated to antibiotic resistance, encodes a thermostable nuclease that aids in the identification of *S. aureus* species (Frickhofen et al., 1990). This gene is commonly used in molecular assays for *S. aureus*. Dai et al. (2021) highlighted the significance of both genes in their study. For precise clinical diagnosis of MRSA, detecting both the *mecA* and *nuc* genes is essential. A single-tube dual-gene detection system can rapidly and accurately identify MRSA strains, streamlining the diagnostic process. Importantly, this system demonstrated a remarkable sensitivity of 10^2^ copies/μL, indicating its significant potential for point-of-care testing (POCT) applications in MRSA detection within clinical settings.

## 2 Materials and methods

### 2.1 RPA primers, gDNA, and probe design

The RPA primers, genomic DNA (gDNA), and probes utilized in this study were customized by GENEWIZ (Suzhou, China) according to the specifications outlined in Supplementary Table S1. The conserved sequences corresponding to the *mecA* (NCBI: BX571856.1) and *nuc* (NCBI: BX571856.1) genes of *S. aureus* were retrieved from the National Center for Biotechnology Information (NCBI) database. Subsequently, the DNA templates were designed using both the TwistAmp^®^ Analysis Design Manual^1^ and Primer Premier 5.0 software. For further details of the DNA design, please refer to Supplementary Table S1. The genomic DNA was tailored to exist as single-stranded DNA featuring a phosphate group at the 5′ end, thereby allowing for complementary base pairing with the amplified target fragment. The design of the gDNA length was based on relevant literature on *Pf*Ago, and a gDNA length of 16 nt was considered sufficient for effective cleavage (Liu et al., 2021). Similarly, the probes were engineered as single-stranded DNA sequences incorporating fluorescent entities such as carboxyfluorescein (FAM) and rhodamine X (ROX) alongside quenching groups, including black hole quenchers 1 or 2 (BHQ1 or −2), positioned at each terminus.

### 2.2 *Pf*Ago expression and purification

The *Pf*Ago gene was synthesized by General Biol (Anhui, China) via codon optimization and subsequently cloned and inserted into the pET-28a (+) vector to enable N-terminal His-tagged *Pf*Ago protein expression in *E. coli* BL21. The bacterial culture was grown in LB media supplemented with 50 μg/mL kanamycin at 37°C and cultured to a volume of 1 L. Once the OD600 reached 0.6–1.0, induction of protein expression was initiated by adding isopropyl-D-thiogalactopyranoside (IPTG), followed by continued incubation at 37°C for 16 h. The bacterial pellet was collected via centrifugation, suspended in lysate, and subjected to ultrasonication on ice to facilitate lysis. The resulting supernatant was then collected by centrifugation at 10,000 × *g* for 20 min at 4°C, followed by affinity chromatography column purification using 50% BeyoGoldTM His-tag Purification Resin. Finally, the purified fractions were analyzed via gel filtration and sodium dodecyl sulfate–polyacrylamide gel electrophoresis.

### 2.3 Construction of plasmids and phylogenetic analysis of *Pf*Ago

The pET28a-6 × His-*Pf*Ago plasmid map of the *Pf*Ago protein (NCBI: WP_011011654.1) was constructed via SnapGene software (Supplementary Table S2A). To investigate the phylogenetic relationship between *Pf*Ago and other characterized Ago proteins, we performed an evolutionary tree analysis using the maximum likelihood method in MEGA-X software on the basis of multiple sequence alignment results. The analysis demonstrated that *Pf*Ago is highly homologous with previously identified thermophilic bacteria (Supplementary Table S2B). This analysis provides valuable insights into the evolutionary history of *Pf*Ago and its potential functional roles.

### 2.4 Preparation of DNA standards

To perform sensitivity analysis, DNA standards were synthesized via *in vitro* transcription. First, the specific sequences of the *nuc* and *mecA* genes of MRSA (ATCC 33591) were amplified via the RPA technique with the primers listed in Supplementary Table S1. The resulting target sequences were then confirmed via agarose gel electrophoresis. After purification, the bacterial genomic DNA was cloned and inserted into the pUC-GW-Kan vector. *In vitro* transcription was conducted via the T7 High-Efficiency Transcription Kit (Beijing Transgenic Biotechnology Co., Ltd., China) following the provided instructions and utilizing the standard plasmid as a template. The concentration of the obtained recombinant plasmid was 300 ng/μL, corresponding to a copy number of 6.0 × 10^10^. The assay template for the RPA-*Pf*Ago assay comprised a 10-fold serial dilution of the transcribed DNA standards, ranging from 10^8^ to 10^10^ copies. Finally, the recombinant plasmids were designated pUC-*nuc* and pUC-*mecA* and employed as research tools for subsequent experiments and analyses.

### 2.5 RPA reaction

The RPA nucleic acid amplification reagent (No. B00000) provided by Hangzhou QiTian Bio-Sci & Tech Co. was used for all procedures. For the RPA amplification system, 25 μL of buffer V, 14.5 μL of deionized water, 2 μL each of forward and reverse primers (10 μM), 4 μL of genomic DNA, and 2.5 μL of Mg^2+^ solution were combined for a total volume of 50 μL. The RPA reaction was conducted at 39°C for 20 min. Subsequently, the amplified fragments were analyzed using 2% agarose gel electrophoresis to screen for suitable primer pairs.

### 2.6 *Pf*Ago detection assays

In a single-gene reaction tube, the following components were added to 20 μL of *Pf*Ago reaction mixture: 2 μL of buffer solution containing 15 mM Tris/HCl (pH 8.0) and 250 mM NaCl, 1.0 μL of MnCl_2_ (40 mM), 4 μL of purified *Pf*Ago (5348.4 μg/mL), 4 μL of complementary amplification products of the *nuc* gene or *mecA* gene with 4 μL of 5′-phosphorylated gDNA (20 μM), 1 μL of ssDNA probe (10 μM), 3 μL of the RPA amplification product, and an appropriate amount of deionized water. In the dual-gene single-tube reaction, the total volume was increased to 35 μL. The reaction mixture included 4 μL of buffer solution, 1.5 μL of MnCl_2_ (40 mM), 7.5 μL of *Pf*Ago, 4 μL of complementary amplification products of the *nuc* gene and *mecA* gene with 8 μL of 5′-phosphorylated gDNA (20 μM), 2 μL of ssDNA probe (10 μM), 3 μL of RPA amplification product, and an appropriate amount of deionized water. The reaction mixture was incubated at 95°C for 30 min. Then, the fluorescence intensity of the FAM/ROX signal was recorded every 30 s using a fluorescence quantitative PCR instrument.

### 2.7 Sensitivity and specificity evaluation

To prepare the two synthesized plasmid standards for detection using the RPA-*Pf*Ago method, the standards were appropriately diluted to achieve copy numbers ranging from 10^1^ to 10^9^ copies/μL. Subsequently, a 10-fold dilution series of the prepared samples was used for sensitivity testing in six replicates. To assess the specificity of the RPA-*Pf*Ago reaction system, we detected the genomes of other pathogens frequently *coinfected with S. aureus* in clinical settings. These pathogens include *Staphylococcus epidermidis* (ATCC 12228), *Staphylococcus hominis* (ATCC 27844), *Staphylococcus saprophyticus* (ATCC 15305), *Staphylococcus hemolyticus* (ATCC 29970), *Pseudomonas aeruginosa* (ATCC 27853), *Acinetobacter baumannii* (ATCC 19606), *Streptococcus pneumoniae* (ATCC 6305), *Clostridium perfringens* (ATCC 13124), and *Klebsiella pneumoniae* (ATCC 13883).

### 2.8 Validation with clinical samples

A total of 40 clinical samples, consisting of 8 wound secretions, 25 sputum samples, and 7 throat swabs, were collected from Chaozhou People’s Hospital. The objective of this study was to assess the feasibility and validate the analytical method of the RPA-*PfA*go system by subjecting these samples to detection using the RPA-*Pf*Ago method. The genomic DNA of MRSA was extracted from these samples using a DNA extraction kit (Beijing TianGen Biotech Co., Ltd.) to serve as a template for the RPA-*Pf*Ago reaction. PCR in combination with agarose gel electrophoresis was then employed to confirm the presence of the *nuc* gene and *mecA* gene in the 40 samples. Additionally, a fully automated bacterial identification system (bioMérieux VITEK 2 Compact) was utilized for pathogen identification, and antimicrobial susceptibility analysis was conducted to evaluate drug sensitivity. Finally, the efficacies of the RPA-*Pf*Ago method and the automated bacterial identification system were compared and assessed.

## 3 Results

### 3.1 The RPA-*Pf*Ago method demonstrates dual recognition and cleavage of *Staphylococcus aureus* and MRSA in molecular biology research

**Figure 1:**
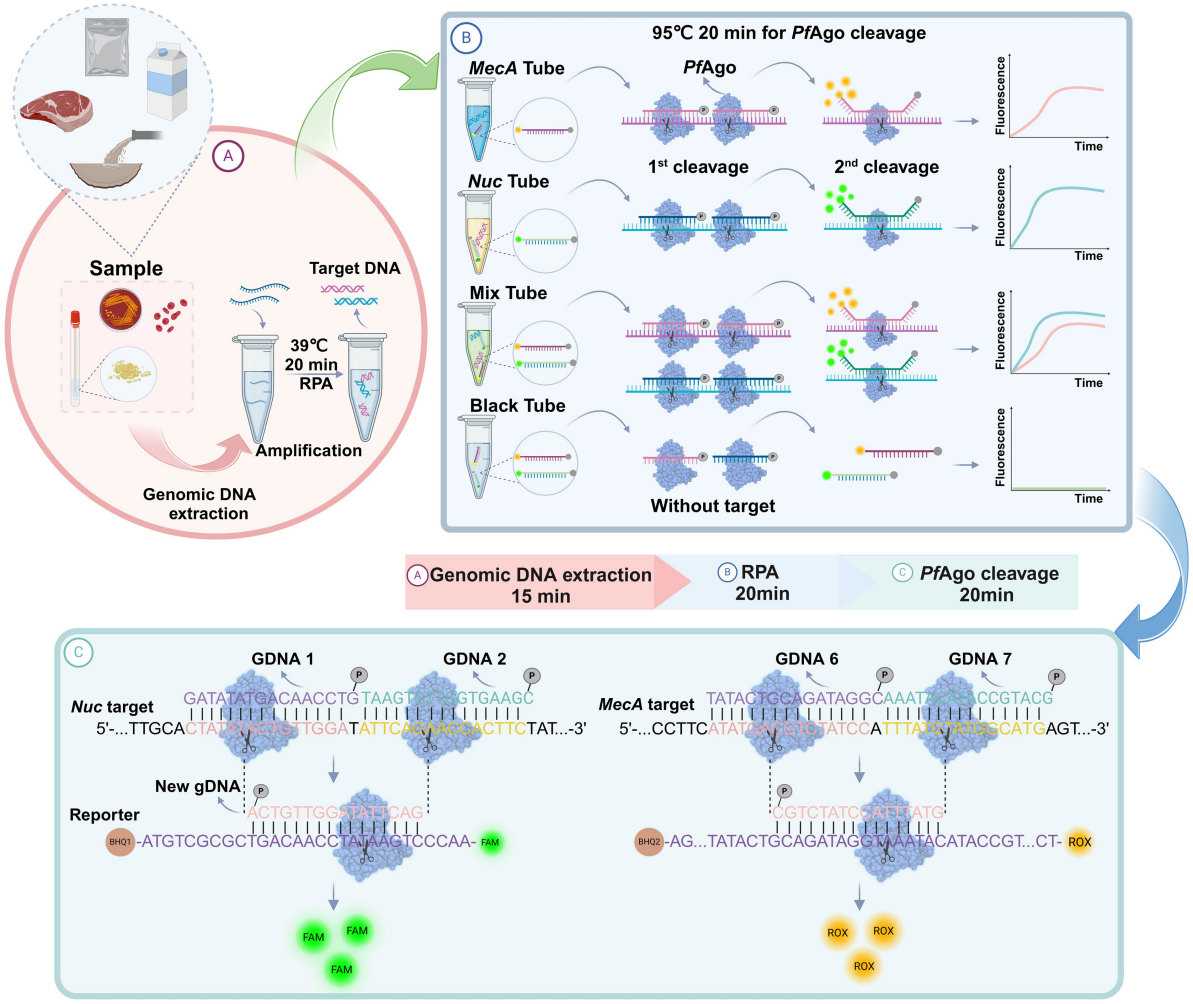
Illustration of the proposed RPA-*Pf*Ago platform for the detection of MRSA. **(A)** Sample extraction DNA and RPA amplification. **(B)** Proposed *Pf*Ago cleavage schematic. **(C)** Cleavage of the *nuc* and *mecA* genes by *Pf*Ago.

As shown in Figure 1, a well-designed assay procedure was developed to fully demonstrate the dual recognition and cleavage properties of *Pf*Ago for *S. aureus* and MRSA. Initially, DNA was extracted from specimens such as swabs, pus, or blood obtained from patients suspected of having *S. aureus* or MRSA infection. The DNA extraction process involved isolating DNA using a preprepared 5% Chelex-100 extract. Subsequently, the RPA-*Pf*Ago assay involved the concurrent amplification of target regions of two genes specific to *S. aureus* and MRSA using RPA at 39°C. The amplified fragments were then specifically identified by the *Pf*Ago protein, which triggers the specific cleavage of the phosphodiester bond between the 10th and 11th bases of the target DNA upon binding to gDNA phosphorylated at the 5′ end (first cleavage). This initial cleavage generated a new 5′ phosphorylated secondary gDNA that acted as a guide for *Pf*Ago to execute a second cleavage. For the second *Pf*Ago cleavage, a single-stranded DNA molecular beacon (MB) was used as the substrate, which emits a fluorescent signal upon cleavage. The specific cleavage of *the nuc* and *mecA* genes by *Pf*Ago resulted in the release of FAM from BHQ1 and ROX from BHQ2. Notably, the *nuc* gene corresponds to FAM, whereas the *mecA* gene corresponds to ROX. This simultaneous detection of both targets can be achieved within a single reaction. To provide a deeper understanding of the underlying mechanism, representative sequences of the amplicon, gDNA, second-cleaved gDNA, and beacon molecules are presented in Figure 1. The current RPA-*Pf*Ago method capitalizes on *Pf*Ago’s specific recognition and cleavage properties, offering dual recognition specificity in contrast to CRISPR-based gene editing techniques.

### 3.2 Specific primer selection, efficient RPA amplification, and fluorescence analysis validated the cleavage efficacy of *Pf*Ago for gene detection

**Figure 2:**
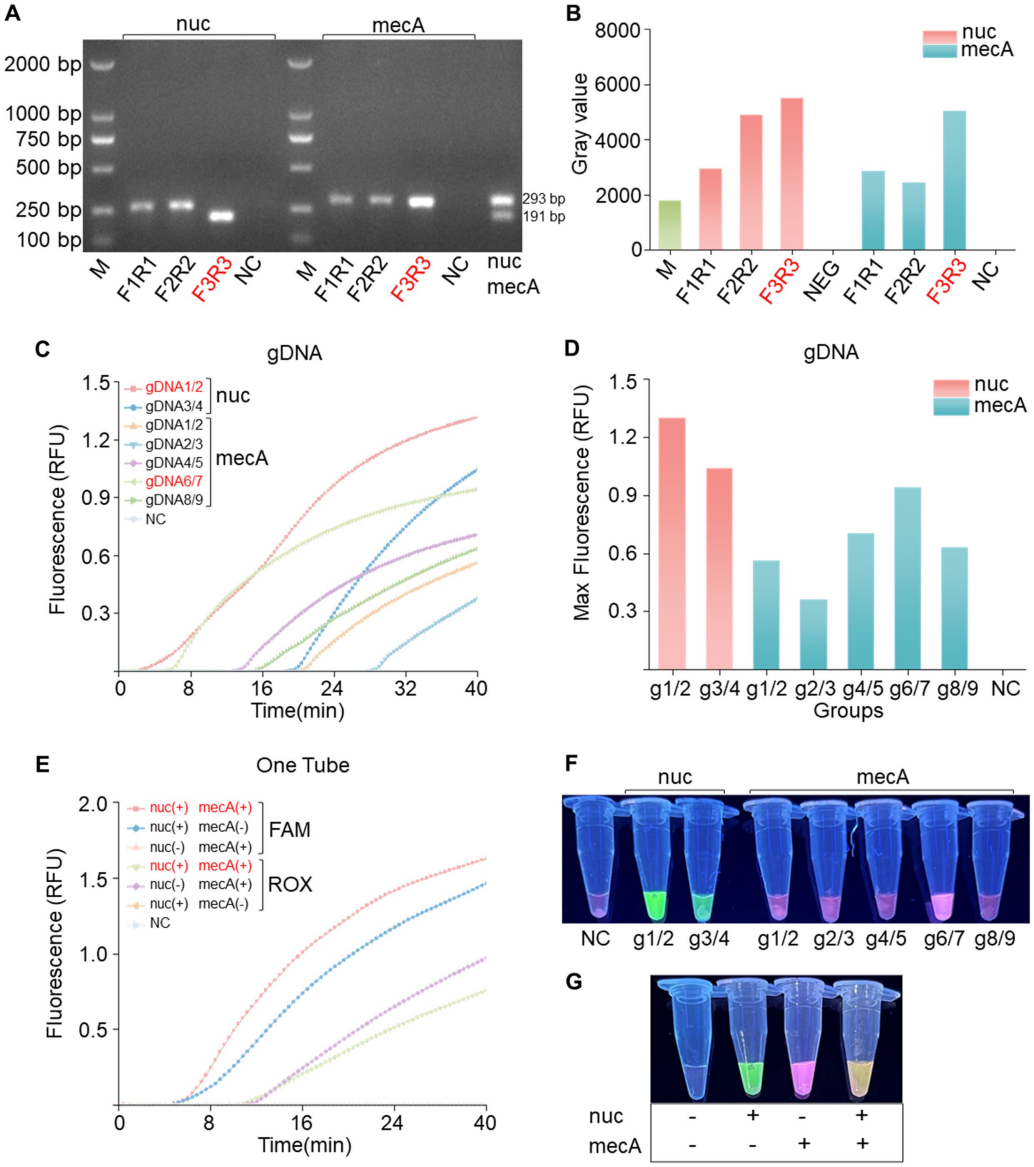
Primer and gDNA screening for single-tube dual-gene reactions. **(A)** Screening of the *nuc* and *mecA* genes for single-gene RPA primers revealed the optimal choice to be the F3R3 primer combination, followed by dual-gene single-tube amplification electrophoresis. **(B)** Analysis of grayscale values from the electrophoresis bands confirmed the superiority of the F3R3 primer combination for both genes. **(C)** Screening for the best gDNA-probe combination for the *nuc* and *mecA* genes included a negative control (NC) using RNase-free water instead of the target plasmid, with RFU indicating relative fluorescence units. **(D)** Bar charts displaying the highest values achieved during screening. **(E)** Evaluation of dual-gene single-tube reactions assessing fluorescence signal intensity for different gene combinations, with a negative control (NC) using RNase-free water in place of the target plasmid. **(F)** Naked-eye visible results showing visually observable outcomes. **(G)** Visualization of fluorescence images corresponding to panel **(E)**.

To achieve effective cleavage by *Pf*Ago in the RPA*-Pf*Ago system, the initial step involves screening specific primer pairs for amplifying target fragments. For each of the two genes, three pairs of RPA primers were meticulously designed. Subsequent gel electrophoresis analysis using a TAE gel consistently revealed bands of the expected sizes. Using the *nuc* gene as an example (Figure 2A), three pairs of primers were used for the RPA reactions. The gel electrophoresis results were then analyzed using ImageJ software (Figure 2B). Notably, the F3R3 primer combination produced the brightest bands, indicating optimal RPA amplification efficiency. Similarly, the *mecA*-F3/*mecA*-R3 combination also exhibited robust amplification. Various combinations of gDNA and probes (*nuc*-g1/2, g3/4; *mecA*-g1/2, g2/3, g4/5, g6/7, g8/9) were devised to guide *Pf*Ago cleavage during the amplification of the two genes. The fluorescence intensity data obtained from the quantitative PCR instrument underscored the superior performance of the *nuc*-gDNA1/2-Probe1 combination, as evidenced by the significantly greater fluorescence intensity than that of the other combinations. A distinct green fluorescence signal was visually observed under blue transillumination. Similarly, the *mecA*-gDNA6/7-Probe3 combination displayed a substantial fluorescence signal (Figures 2C,D,F). In conclusion, the fluorescence intensity results confirmed the efficacy of the RPA-*Pf*Ago method for detecting the *nuc* and *mecA* genes, highlighting its potential utility in diagnosing bacterial infections.

### 3.3 The novel *Pf*Ago-based system shows specific fluorescence in single-gene amplification and a dual response in cleaving both genes

In the previous step, we carefully selected optimal gDNA–Probe combinations tailored for individual gene detection of the *nuc* and *mecA* genes. Building upon this foundation, our study introduces a novel dual gene detection system. Our experimental findings unequivocally revealed that each gene, when individually amplified, elicited a specific fluorescence response, producing distinct fluorescence signals without any instance of cross-reactivity (Figures 2E,G). Intriguingly, upon simultaneous *Pf*Ago cleavage of both genes, a dual fluorescence phenomenon emerged, manifesting as a visually striking pale-yellow fluorescence. This dual signal comprised a green component from FAM and a red component from ROX. These results, characterized by the absence of cleavage reactions in the absence of *Pf*Ago, serve as definitive confirmation of our methodology.

### 3.4 Optimization of the RPA-*Pf*Ago reaction involved adjusting the Mn^2+^, probe, and *Pf*Ago concentrations for dual-gene cleavage

**Figure 3:**
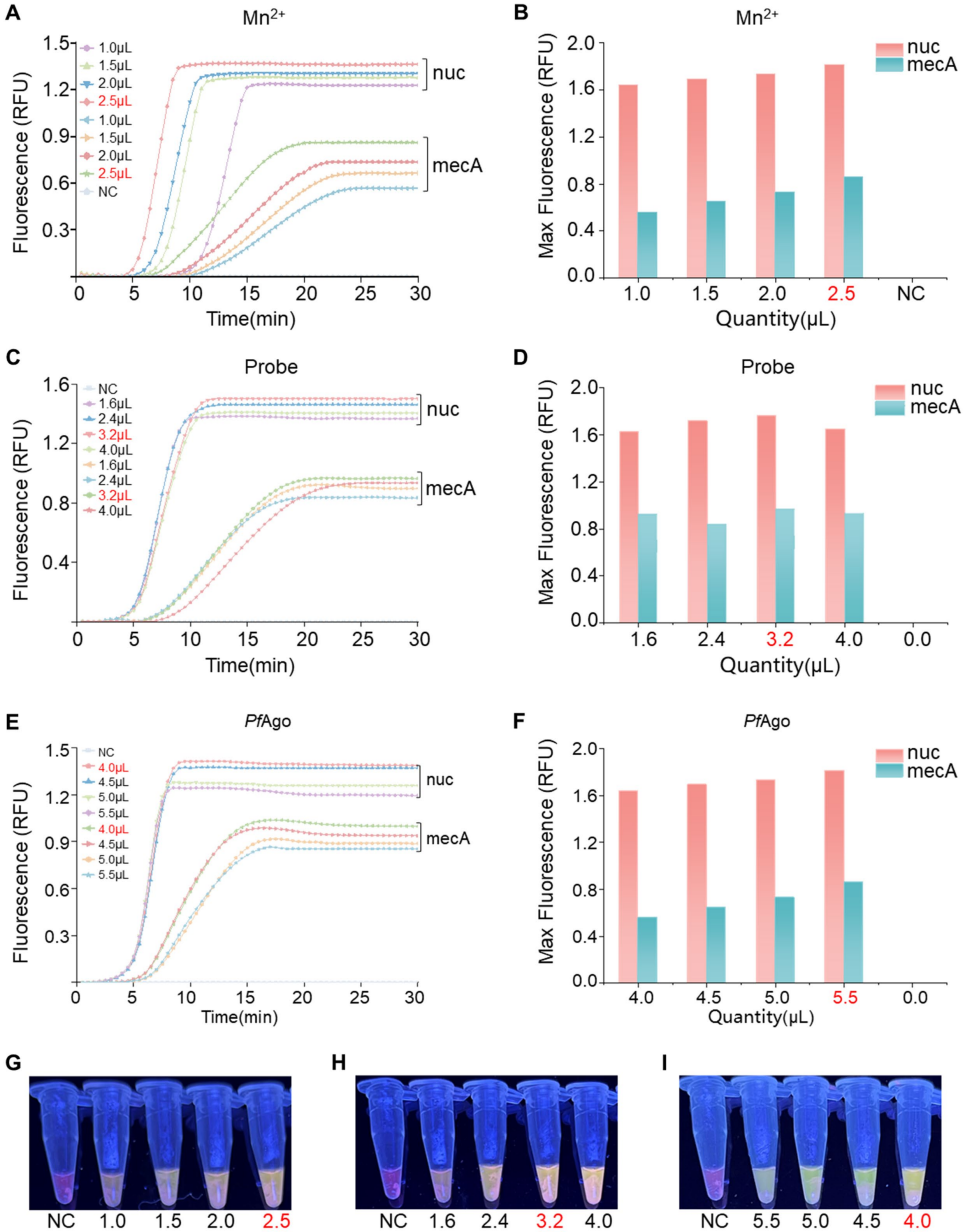
Optimization of the RPA-*Pf*Ago system. **(A)** Evaluation of enzyme cleavage fluorescence signals in the dual-gene reaction system with varying Mn^2+^ concentrations. **(B)** Representation of the maximum fluorescence signal achieved with different Mn^2+^ concentrations in a bar chart. **(C)** Assessment of enzyme cleavage fluorescence signals in the dual-gene reaction system with diverse probe concentrations. **(D)** Display of the maximum fluorescence signal obtained with varying probe concentrations in a bar chart. **(E)** Examination of enzyme cleavage fluorescence signals in the dual-gene reaction system with different levels of *Pf*Ago. **(F)** Demonstration of the maximum fluorescence signal intensity of *Pf*Ago at different concentrations in a bar chart. **(G)** Observation of visible blue transmission to the naked eye from the Mn^2+^ tubes. **(H)** Observation of visible blue transmission through the probe tubes to the naked eye. **(I)** Observation of visible blue transmission through the *Pf*Ago tubes to the naked eye, with RNase-free water used as the negative control (NC) instead of the target plasmid. RFU, relative fluorescence units.

An optimization analysis was performed to determine the ideal reaction system for RPA-*Pf*Ago by adjusting the concentrations of key components (Mn^2+^, probe, and *Pf*Ago) in the dual-gene reaction setup. Initially, different Mn^2+^ concentrations (1.0, 1.5, 2.0, and 2.5 μM; Figures 3A,B,G) were assessed, with the highest enzyme cleavage efficiency observed at 2.5 μM Mn^2+^. Subsequently, the influence of varying probe concentrations on both genes was investigated (*nuc*: 1.0, 1.4, 1.8, and 2.2 μM; *mecA*: 1.0, 1.4, 1.8, and 2.2 μM; Figures 3C,D,H), revealing optimal concentrations of 1.4 μM for *nuc* and 1.8 μM for *mecA*. Finally, different *Pf*Ago concentrations were tested (4.0, 4.5, 5.0, and 5.5 μL; Figures 3E,F,I), and the results demonstrated that 4 μL of *Pf*Ago yielded the most effective results.

### 3.5 The RPA-*Pf*Ago method detects gene concentrations as low as 10^2^ copies/μL and shows no cross-reactivity

**Figure 4:**
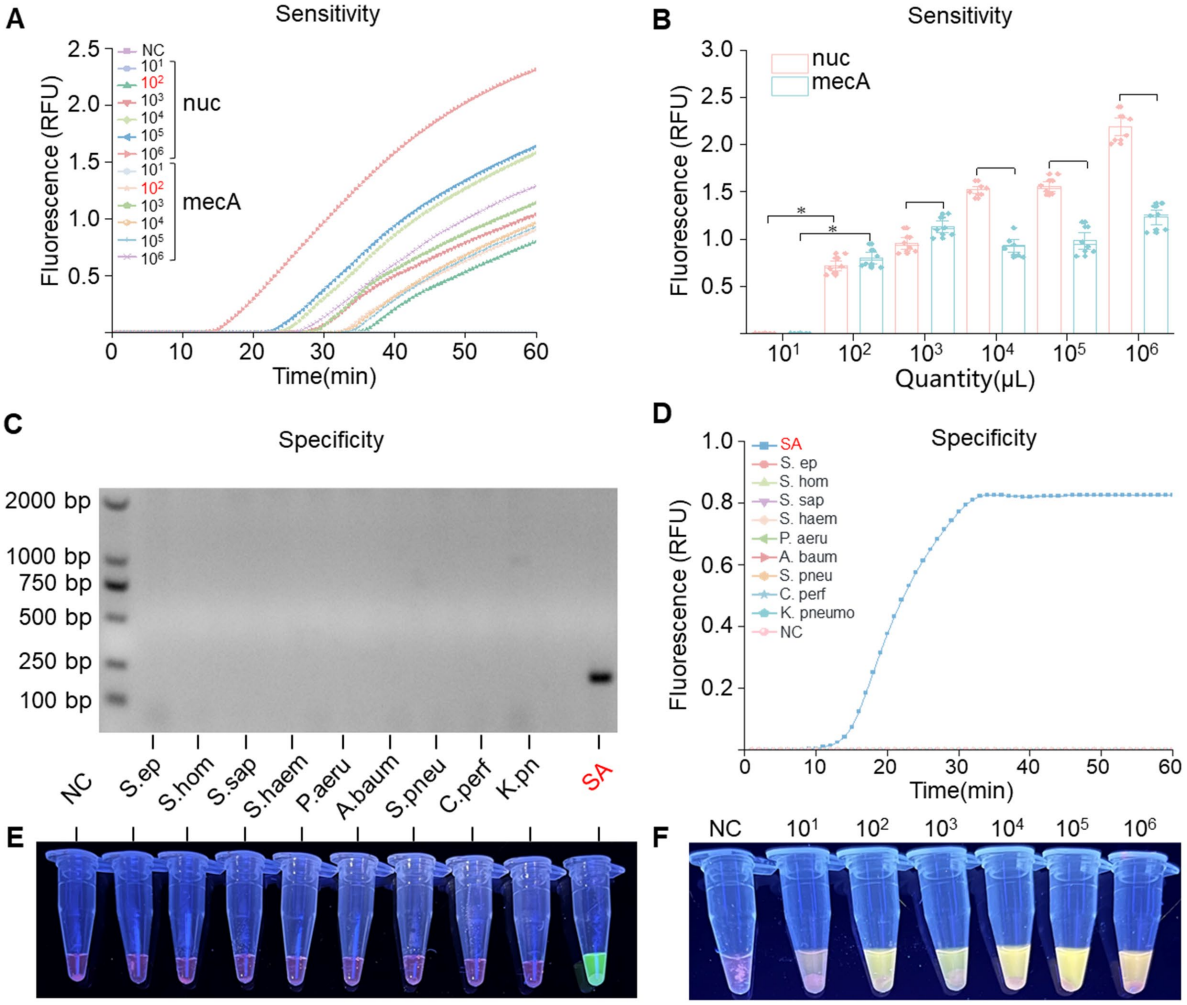
Sensitivity and specificity assessment of the RPA-*Pf*Ago system. **(A)** Real-time fluorescence signals monitored from 0 to 60 min posttreatment with various concentrations of the target plasmid. **(B)** Determination of the limit of detection for RPA-*Pf*Ago detection on standard MRSA plasmids (*p < 0.0001, *n* = 6). **(C)** Visualization of the RPA reaction through agarose gel electrophoresis. **(D)** Evaluation of RPA-*Pf*Ago sensitivity indicated by the blue transmittance of fluorescent tubes. **(E)** Illustration of RPA-*Pf*Ago specificity demonstrated by the blue transmittance of fluorescent tubes. **(F)** Illustration of the sensitivity of the RPA-*Pf*Ago dual detection by the blue transmittance of fluorescent tubes.

To assess the sensitivity of RPA-*Pf*Ago, recombinant plasmids containing the *nuc* and *mecA* genes were diluted using a gradient approach, resulting in template concentrations ranging from 10^1^ to 10^9^ copies/μL. By analyzing the fluorescence signals, we determined that the RPA-*Pf*Ago system could detect the *nuc* gene at concentrations as low as 10^2^ copies/μL and the *mecA* gene at concentrations as low as 10^2^ copies/μL (Figures 4A,F), with consistent results observed in three repeated experiments, demonstrating good stability. The inverse relationship between substrate concentration and RFU, as illustrated in Figure 4B, may indicate the occurrence of substrate inhibition, a phenomenon where excessive substrate molecules compete for the active sites of the enzyme, thereby reducing its catalytic efficiency. To evaluate the specificity of the RPA-*Pf*Ago system, we tested the genomes of other pathogenic bacteria using this method, and the results indicated no cross-reactivity (Figures 4D,E). Additionally, gel electrophoresis was performed to confirm the presence of the RPA products, which confirmed the presence of *S. aureus* exclusively (Figure 4C).

### 3.6 The optimized RPA-*Pf*Ago system accurately detected MRSA in clinical samples with 95.0% accuracy

**Figure 5:**
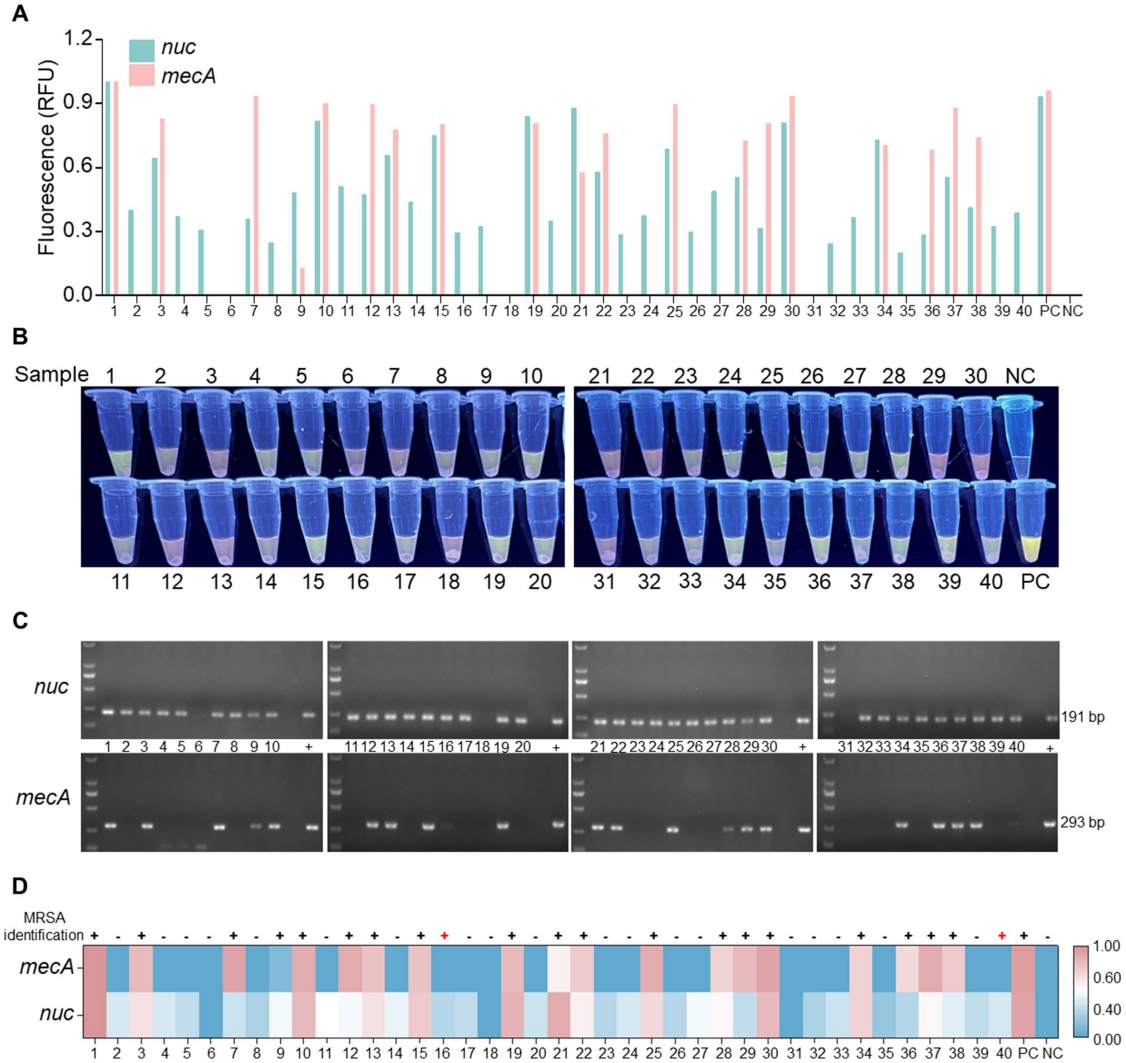
Analysis of clinical samples utilizing the RPA-*Pf*Ago system. **(A)** Fluorescence signal of the *nuc* and *mecA* genes observed at the endpoint of the RPA-*Pf*Ago system reaction. Standard plasmids were used as positive controls. **(B)** Visualization of the mixed tube reaction through the blue transmittance of fluorescent tubes. **(C)** Examination of the *nuc* and *mecA* genes through agarose gel electrophoresis following PCR. **(D)** Comparative analysis of clinical samples tested using the vitek2-compact drug sensitivity detection system and the RPA-*Pf*Ago method.

To evaluate the clinical detection performance of the optimized RPA-*Pf*Ago detection system, 40 clinical samples were tested. Among the 40 samples examined, 22 were found to contain either the *nuc* or *mecA* gene. The detection was carried out visually with the naked eye under UV light using RPA-*Pf*Ago (Figures 5A,B). Subsequently, 22 samples were confirmed to be MRSA, 38 samples tested positive for the *nuc* gene, and 2 samples tested negative. The agarose gel electrophoresis results for the *nuc* and *mecA* genes supported the efficacy of the RPA-*Pf*Ago method (Figure 5C). Additionally, suspected strains were further identified using bacterial culture methods and fully automated bacterial identification (bioMérieux VITEK 2 Compact) along with antimicrobial susceptibility analysis (Supplementary Table S4), resulting in an accuracy rate of 95.0% (Figure 5D). The discrepancy observed in the results could be attributed to prior clinical detection processes that might have reduced the bacterial load in the samples. These findings underscore the comparable performance between the RPA-*Pf*Ago detection system and conventional clinical identification methods, highlighting its ability to accurately, rapidly, and simultaneously identify target genes critical for early MRSA infection diagnosis and spread control. However, challenges remain in terms of sensitivity compared with established clinical detection techniques, necessitating further optimization for broader clinical diagnostic applications.

## 4 Discussion

In hospital settings, MRSA infections are prevalent and pose significant risks of severe complications and mortality (Azzam et al., 2023), particularly among immunocompromised patients. Rapid and accurate detection of MRSA is essential for infection control in hospital settings (Calfee et al., 2014). Detection depends on phenotypic characteristics determined by the *mecA* gene, such as elevated minimum inhibitory concentrations against beta-lactam antibiotics or diminished inhibition zones for oxacillin and cefoxitin (Brown et al., 2005). Accurate, swift, and commercially available PCR methods represent the gold standard in hospital settings for detecting the *mecA* and *nuc* genes (Rajan et al., 2007). However, their widespread application is restricted by high costs and complex equipment requirements, especially in resource-limited settings. Moreover, conventional culture techniques typically require multiple days of incubation on agar plates, followed by additional biochemical tests to verify the presence of *S. aureus*, thereby increasing the risk of false positives (Towner et al., 1998) and potentially prolonging the diagnostic and therapeutic process. Thus, there is an urgent need to develop swift and precise detection methods to effectively manage MRSA infections in clinical settings.

*Pf*Ago is an enzyme–protein complex that binds short gDNA molecules for specific target recognition and cleavage (Swarts et al., 2015), enabling rapid detection of multiple pathogenic gene targets. Combining the high specificity of *Pf*Ago for particular DNA sequences with the efficient isothermal amplification technique RPA has led to the recent application of *Pf*Ago-RPA hybrids in various pathogen detection methods (Yang et al., 2023; Liu et al., 2023b; Lin et al., 2024). Moreover, the RPA-*Pf*Ago detection method has demonstrated applicability not only in medical settings but also in fields such as food safety for pathogen detection (Lin et al., 2024). Notably, programmable nucleases such as CRISPR/Cas have been widely reported and applied in the detection field (He et al., 2022; Zhou et al., 2024). *Pf*Ago not only shares programmability, sequence specificity, and high sensitivity akin to those of CRISPR/Cas but also has limitations, such as dependency on PAM sequences and nonspecific transactivation cleavage, which restrict the use of multiplex CRISPR/Cas in single-tube assays. Here, we established a single-tube RPA-*Pf*Ago detection platform for detecting the MRSA *nuc* and *mecA* genes. To prevent aerosol contamination during the reaction, the RPA amplification system was added to the bottom of the reaction tube, while the *Pf*Ago cleavage system was added to the lid of the tube. Following the completion of the RPA amplification reaction, a simple centrifugation step mixes the RPA and *Pf*Ago detection systems. Thus, the single-tube RPA-*Pf*Ago detection platform minimizes aerosol contamination and achieves rapid and sensitive detection of MRSA.

The *Pf*Ago assay allows the detection of multiple targets in a single reaction. In the surveillance of patient-specific bacterial pathogens, the concomitant measurement of pathogen-encoded resistance genes is of paramount importance. The absence of timely detection of these resistance genes could lead to a missed window of optimal therapeutic intervention, thereby escalating the risks to the patient. For example, the RPA combined with an orthogonal CRISPR dual system has been utilized for single-tube detection of the genes, *tcdA* and *tcdB* (Jiang et al., 2023). Various types and quantities serve clinical POCTs more effectively. Thus, compared with repeated singleplex detection, the use of a multiplex detection system can be advantageous in minimizing the volume of reagents and genomic samples needed. In this study, we confirmed the sequence-specific cleavage activity of RPA-*Pf*Ago, with a particular focus on the formation of secondary gDNA-*Pf*Ago complexes and the cleavage of the reporter gene, allowing a mixture of various sequences and multiple reporter genes in a single detection event. This unique feature enables us to design a duplex RPA-*Pf*Ago assay targeting the *nuc* and *mecA* genes. Moreover, given the availability of various dyes with different excitation wavelengths and instruments capable of recognizing these dyes, the number of targets in RPA-*Pf*Ago detection can be increased. For example, Li et al. (2023) developed a pentaplex detection method using FAM, VIC, CY5, TAMRA, and ROX dyes along with an RT–qPCR system for the simultaneous detection of hepatitis B virus, hepatitis C virus, hepatitis E virus, *Treponema pallidum*, and the RNase P gene. Future research should focus on the design and demonstration of RPA-*Pf*Ago assays for the integration of multiple targets.

For rapid detection, we utilized a 25 μL RPA-*Pf*Ago system for amplification, with a reaction time of 55 min. Compared with the PCR-*Pf*Ago, LAMP-*Pf*Ago, and RPA-CRISPR methods, the RPA-*Pf*Ago method significantly reduced the reaction time, enhancing the on-site detection efficiency (Table 1). Furthermore, we observed that the sensitivity of RPA-*Pf*Ago was greater than that of other molecular detection methods. Nonetheless, the RPA-*Pf*Ago assay, which has a sensitivity of 10^2^ copies/μL, encounters a detection limitation when juxtaposed with DNAzyme-*Pf*Ago (Li et al., 2024), which has a superior sensitivity of 1 CFU/mL. They can be combined in a standardized and coordinated way to compensate for their respective shortcomings and offer more reference methods for MRSA. Notably, at elevated substrate concentrations, the fluorescence signal may be compromised by quenching effects, potentially masking the detection of increased product formation and consequently leading to reduced relative fluorescence unit (RFU) measurements. We suggest that the calibration of a fluorescence detection system with a dynamic range that accommodates high substrate concentrations is necessary to accurately quantify the fluorescence signal and to avoid potential underestimations. Notably, the development of an RPA-*Pf*Ago assay involves the design of two RPA primers, a reporter, and two gDNAs, which are more complex than PCR-based assays. Therefore, future research can focus on improving the LOD and providing quantitative measurements to accurately detect MRSA in clinical samples. Additionally, this approach has limitations in terms of the stringent storage conditions required for the *Pf*Ago protein. Future research could explore leveraging *Pf*Ago’s DNA-specific recognition properties through integration with microfluidic chip technology (Shen et al., 2023; Xiu et al., 2023), quartz crystals (Wang and Yang, 2024) or field-effect transistors (Kong et al., 2024) to achieve high-throughput automated detection. Additionally, addressing these challenges could include improving the protein structure (Fu et al., 2019) or enhancing its purity. Overall, this technology not only opens new avenues for rapid and reliable pathogen detection in microbial infection management but also presents broader prospects in molecular diagnostics.

**Table 1:** Molecular methods for MRSA detection.

Methods	Detection time (min)	LOD	Signal readout	References
ddPCR	>66	4 × 10^3^ cfu/mL	Fluorescence	Kelley et al. (2013)
PCR-LFI	78	20 fg	Test strip	Zhang et al. (2017)
Multiplex RT-PCR	35	10^3^ cfu/mL	Fluorescence	Wang et al. (2014)
LAMP-*Pf*Ago	65	1 cfu/mL	Fluorescence	Li et al. (2024)
RPA-*Pf*Ago	55	10^2^ copies/μL	Fluorescence	This-study
Cas9	>60	10 cfu/mL	Fluorescence	Guk et al. (2017)
PCR-Cas14a	>120	1.23 ng/mL	Fluorescence	Tao et al. (2023)
Cas9n-Cas12a	170	1 cfu/mL	Fluorescence	Hu et al. (2023)
PCR	120	1 pg	Electrophoresis	Duraiswamy et al. (2023)
RT-PCR	65	–	Fluorescence	Sahibzada et al. (2020)
LAMP	75	–	Fluorescence	Chen et al. (2017)
RPA-CRISPR	135	8 cfu/mL	Fluorescence	Wei et al. (2022)

ddPCR, droplet digital PCR; LFI, lateral flow immunoassay; LAMP, loop-mediated isothermal amplification; LCR, ligase chain reaction; RPA, recombinase polymerase amplification; Cas, CRISPR-associated proteins; RT-PCR, reverse transcription-polymerase chain reaction.

## Data Availability

The datasets presented in this study can be found in online repositories. The names of the repository/repositories and accession number(s) can be found in the article/Supplementary material.

## Footnotes

^1^ https://www.twistdx.co.uk/resources/twistamp-analysis-design-manual
