# Supplementary material for: Rapid and sensitive detection of methicillin-resistant Staphylococcus aureus through the RPA-PfAgo system
Source: Front Microbiol. 2024 Aug 21;15:1422574. doi: 10.3389/fmicb.2024.1422574 (PMC11371615; doi:10.3389/fmicb.2024.1422574)
Supplement: Supplementary file 1 [file Data_Sheet_1.docx]

**Rapid and Sensitive Detection of Methicillin-Resistant *Staphylococcus aureus* through the RPA-*Pf*Ago System**

Weizhong Chen^a^, Jiexiu Zhang^b^, Huagui Wei^c^, Jie Su^d^, Jie Lin^a^, Xueyan Liang^e^, Jiangtao Chen^e^, Rong Zhou^a^, Lin Li^a^, Zefang Lu^a^, Guangyu Sun^a, *^

a Chaozhou People's Hospital, Shantou University Medical College, Chaozhou, China.

b Department of Histology and Embryology, Shantou University Medical College, Shantou, China.

c School of Laboratory Medicine, Youjiang Medical University for Nationalities, Baize, China.

d Chaozhou Central Hospital, Laboratory Department, Chaozhou, China.

e Huizhou Central Hospital, Laboratory Department, Huizhou, China.

* Corresponding author.

**Table S1:** The sequence of four oligonucleotides about S. aureus used in RPA.

| Name | Sequence (5’-3’) | Description |
| --- | --- | --- |
| nuc-F1 | GTAGCTCAGCAAATGCATCACAAACAGA | Primers targeting *S.aureus* and *MRSA* genes |
| nuc-R1 | TTTTTCGTAAATGCACTTGCTTCAGGAC |  |
| nuc-F2 | GTCTAAGTAGCTCAGCAAATGCATCACA |  |
| nuc-R2 | TTTCGTAAATGCACTTGCTTCAGGACCA |  |
| nuc-F3 | GTAGCTTCAAGTCTAAGTAGCTCAGCAAAT |  |
| nuc-R3 | GGTGTATCAACCAATAATAGTCTGAATGTC |  |
| mecA-F1 | ACTTGTTGAGCAGAGGTTCTTTTTTATC |  |
| mecA-R1 | CTATCGTGTCACAATCGTTGACGATAAT |  |
| mecA-F2 | TGAGCAGAGGTTCTTTTTTATCTTCGGT |  |
| mecA-R2 | AGATGGCTATCGTGTCACAATCGTTGAC |  |
| mecA-F3 | GAGCAGAGGTTCTTTTTTATCTTCGGTT |  |
| mecA-R3 | ATGGCTATCGTGTCACAATCGTTGACGA |  |
| nuc-gDNA 1 | P-GTCCAACAGTATATAG | gDNA and molecular markers associated with the *Pf*Ago cleavage system |
| nuc-gDNA 2 | P-CGAAGTGGTTCTGAAT |  |
| nuc-Probe 1 | FAM-AACCCTGAATATCCAACAGTCGCGCTG  TA-BHQ1 |  |
| nuc-gDNA 3 | P-GAAGCGATTGATGGTG |  |
| nuc-gDNA 4 | P-GCCTGCGACATTAATT |  |
| nuc-Probe 2 | FAM-ACTGTTAATTAAAGCGATTGCGTTCTCG  C-BHQ1 |  |
| mecA-gDNA 1 | P-GCTCATGCCATACATA |  |
| mecA-gDNA 2 | P-GATGGATAGACGTCAT |  |
| mecA-gDNA 3 | P-CTGAAGGTGTGCTTAC |  |
| mecA-gDNA 4 | P-CGCCATACATAAATGG |  |
| mecA-gDNA 5 | P-CTAGACGTCATATGAA |  |
| mecA-gDNA 6 | P-CGGATAGACGTCATAT |  |
| mecA-gDNA 7 | P-GCATGCCATACATAAA |  |
| mecA-gDNA 8 | P-GATAAATGGATAGACG |  |
| mecA-gDNA 9 | P-GCATATGAAGGTGTGC |  |
| mecA-Probe 3 | ROX-TCATGCCATACATAAATGGATAGACGTC  ATATGAAGGTGTTGGCATGA-BHQ2 |  |

**Table S2:** Expression and purification of *Pf*Ago. (A) Schematic map of the pET 28a-6×His-*Pf*Ago plasmid for recombinant expression of *Pf*Ago. (B) Evolutionary tree analysis using maximum likelihood method between *Pf*Ago and other characteristic Ago proteins. (C) Analysis of the protein samples at different stages of purification by SDS-PAGE. (D) Concentration range of *Pf*Ago indicated by gradient dilution of bovine serum albumin.


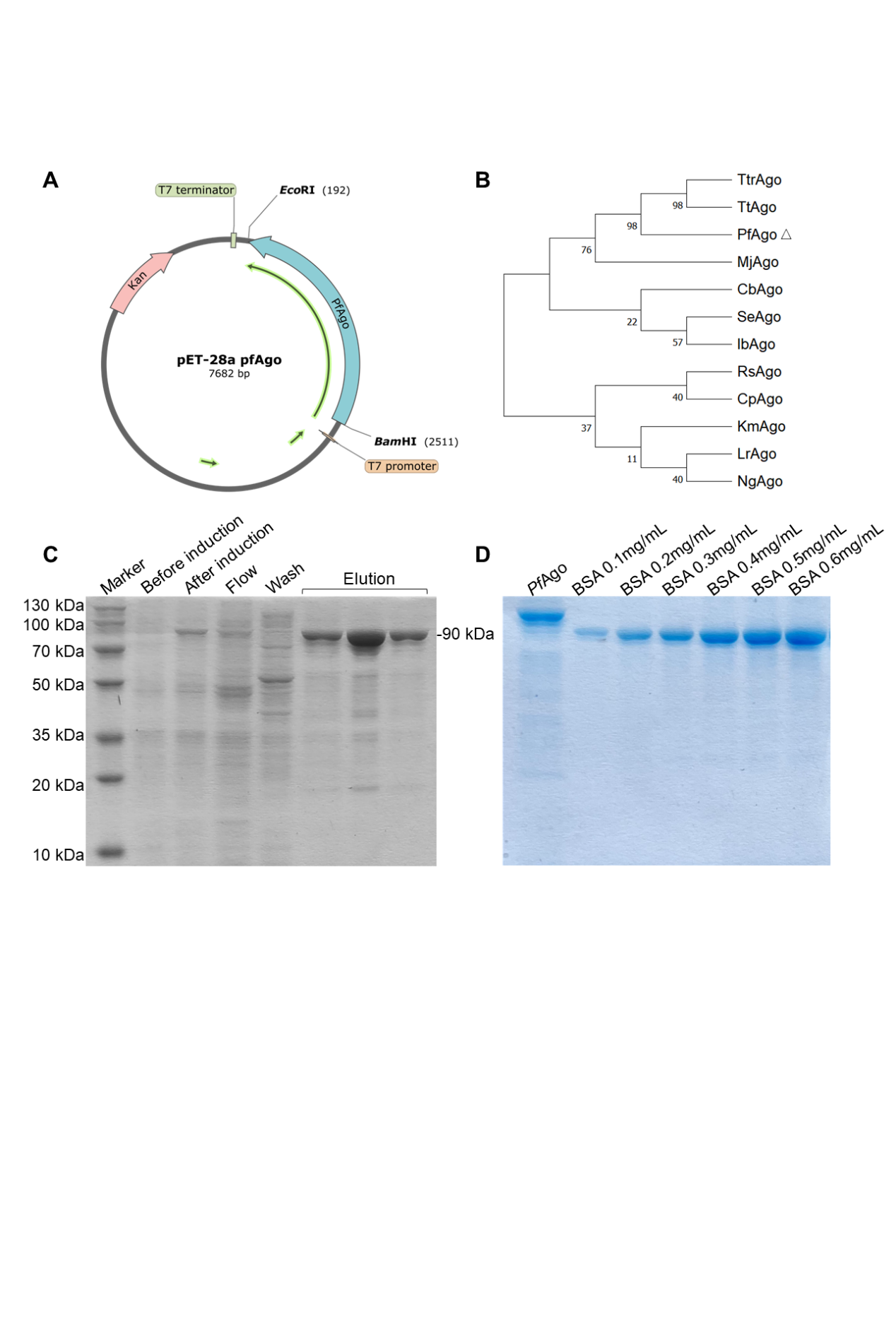


**Table S3:** The sequence of mock template used in *Pf*Ago cleavage system.

| Name | Sequence (5’-3’) | Description |
| --- | --- | --- |
| *Nuc* ssDNA  (191bp) | GTAGCTTCAAGTCTAAGTAGCTCAGCAAATGCATCACAAACAGATAATGGCGTAAATAGAAGTGGTTCTGAATATCCAACAGTATATAGTGCAACTTCAACTAAAAAATTACATAAAGAACCTGCGACATTAATTAAAGCGATTGATGGTGATACTGTTAAATTAATGTACAAAGGTCAACCAATGACATTCAGACTATTATTGGTTGATACACC | ssDNA about *nuc* (NCBI: BX571856.1, 895808-896022 |
| *MecA* ssDNA  (293bp) | GAGCAGAGGTTCTTTTTTATCTTCGGTTAATTTATTATATTCTTCGTTACTCATGCCATACATAAATGGATAGACGTCATATGAAGGTGTGCTTACAAGTGCTAATAATTCACCTGTTTGAGGGTGGATAGCAGTACCTGAGCCATAATCATTTTTCATGTTGTTATAAATACTCTTTTGAACTTTAGCATCAATAGTTAGTTGAATATCTTTGCCATCTTTTTTCTTTTTCTCTATTAATGTATGTGCGATTGTATTGCTATTATCGTCAACGATTGTGACACGATAGCCAT | ssDNA about *mecA* (NCBI: BX571856.1,45750-46042) |

**Table S4:** The information about the clinical samples.

| Number | Gender | Age | Drugsen sitivity results |
| --- | --- | --- | --- |
| S. aureus *clinical* samples | | | |
| #1 | female | 45 | positive |
| #2 | male | 34 | negative |
| #3 | male | 41 | negative |
| #4 | male | 57 | positive |
| #5 | male | 36 | negative |
| #6 | female | 44 | negative |
| #7 | male | 59 | positive |
| #8 | female | 54 | negative |
| #9 | male | 38 | positive |
| #10 | male | 48 | negative |
| #11 | female | 80 | positive |
| #12 | male | 47 | negative |
| #13 | female | 78 | positive |
| #14 | female | 65 | positive |
| #15 | male | 57 | positive |
| #16 | male | 47 | negative |
| #17 | female | 64 | positive |
| #18 | male | 57 | negative |
| #19 | female | 69 | positive |
| #20 | male | 34 | negative |
| #21 | male | 61 | positive |
| #22 | male | 67 | positive |
| #23 | female | 85 | positive |
| #24 | male | 55 | negative |
| #25 | male | 58 | negative |
| #26 | female | 73 | positive |
| #27 | male | 79 | positive |
| #28 | female | 63 | positive |
| #29 | female | 28 | negative |
| #30 | male | 74 | positive |
| #31 | male | 63 | positive |
| #32 | female | 87 | positive |
| #33 | male | 52 | negative |
| #34 | female | 49 | negative |
| #35 | male | 49 | positive |
| #36 | male | 57 | negative |
| #37 | male | 60 | positive |
| #38 | male | 59 | negative |
| #39 | female | 47 | negative |
| #40 | male | 84 | positive |
